# “Am I Teaching into the Void?” Instructor Perceptions of Active Learning in Online Biology Courses

**DOI:** 10.1093/iob/obag053

**Published:** 2026-09-18

**Authors:** Austin L Zuckerman, David Esparza, Yanwei C Liu, Michelle K Smith

**Affiliations:** Department of Ecology and Evolutionary Biology, Cornell University, Ithaca, NY 14853, USA; Department of Ecology and Evolutionary Biology, Cornell University, Ithaca, NY 14853, USA; Department of Physics, Drexel University, Philadelphia, PA, USA; Department of Ecology and Evolutionary Biology, Cornell University, Ithaca, NY 14853, USA; Department of Ecology and Evolutionary Biology, Cornell University, Ithaca, NY 14853, USA

## Abstract

Extensive studies and meta-analyses have shown that active learning in science, technology, engineering, and mathematics courses improve equitable learning outcomes compared to traditional lecture. Before the COVID-19 pandemic, most active learning studies focused on in-person instruction, but as online and hybrid teaching continues to be explored as a potential avenue to deliver course content to more students, the field is exploring the efficacy of active learning practices in online courses. Several studies have compared student course performance and affective dispositions toward active learning practices between online and in-person courses, yet there has been limited work examining experiences from the instructor’s perspective. The future of online teaching is at a critical juncture as institutions decide whether and how to scale online biology instruction in ways that are sustainable for instructors while conferring positive learning outcomes for all students. In this study, we surveyed a national sample of higher education biology instructors who taught both in-person and online biology courses using active learning. This survey compares instructor experiences using active learning in their most recently taught online and in-person biology courses. We also asked instructors about insights into barriers and relative advantages of implementing active learning in online biology courses. Instructors perceived active learning to be aligned with their preferred pedagogical styles in both in-person and online courses, yet they viewed active learning implementation significantly more positively for their in-person courses. Instructors perceived that there was weaker departmental support and visible resources for supporting active learning in their online courses. The reported barriers for using active learning in online courses largely centered around student engagement and buy-in, and active learning strategies that worked better in the online courses were typically attributed to structural advantages of the activities in the online modality rather than pedagogical innovation. Informed by advice curated from the participant sample, we provide recommendations that focus on opportunities to improve active learning in online biology courses.

## Introduction

It has been more than a decade since the publication of a landmark meta-analysis documenting the outcomes conferred by active learning practices in undergraduate science, technology, engineering, and mathematics (STEM) education (Freeman et al. 2014). This publication sparked national conversations and research studies on disseminating these practices more ubiquitously in higher education STEM courses (e.g., Stains et al. 2018; Theobald et al. 2020; Apkarian et al. 2021; Sandrone et al. 2021). In a national survey of biology education researchers and instructors and a review of journal articles, Driessen et al. (2020) developed the following working definition of active learning: “*Active learning is an interactive and engaging process for students that may be implemented through the employment of strategies that involve metacognition, discussion, group work, formative assessment, practicing core competencies, live-action visuals, conceptual class design, worksheets, and/or games*.” Grounded in this definition, active learning pedagogy emphasizes a student-centered learning model that has been recommended by *Vision and Change in Undergraduate Biology Education* (American Association for the Advancement of Science 2011), an initiative that has united the biology education community toward a shared vision on curricular and pedagogical reform.

Active learning strategies are a mechanism for reorienting classrooms to be more student-centered by shifting the role of the instructor from passively transmitting information to scaffolding students’ metacognitive strategies so that they become more self-regulated learners (AAAS 2011; Armbruster et al. 2009; Connell et al. 2016). This reorientation is grounded in constructivist learning theory, which posits that learners are not “blank slates,” and knowledge formation is an active and reflective process where learners construct meaning and modify their conceptual models of phenomena through interactive and immersive activities (Pugh et al. 2010; von Glasersfeld 1990). Active learning practices aligned with constructivist theory have been shown to improve student critical thinking (e.g., Knight and Wood 2005; Nelson and Crow 2014; Holmes et al. 2015; Styers et al. 2018), conceptual understanding of core biological concepts (e.g., Olimpo and Esparza 2020; Nehm et al. 2022), and learning outcomes compared to traditional lecture-style courses, notably resulting in decreased failure rates and a reduction in equity gaps for minoritized student groups in STEM courses (Knight and Wood 2005; Deslauriers et al. 2011; Eddy and Hogan 2014; Freeman et al. 2014; Theobald et al. 2020). Active learning has also been shown to promote positive student affect toward their curricular experiences (Armbruster et al. 2009) and scientific interest (Owens et al. 2020).

Implementation of active learning has implications for teaching practice (Dewsbury and Brame 2019), and there is less of a question as to *whether* these practices are beneficial but instead *how* these practices can become more widely adopted to improve student outcomes. Importantly, the value of implementing different active learning techniques and strategies depends on whether activities place metacognitive responsibility onto students rather than simply making students procedurally active. That is, not all activities where students are active participants are beneficial to learning, especially activities that lack constructivist elements. For example, activities that only engage students in lower cognitive tasks such as recall, or misconception-focused instruction that fails to leverage and address students’ prior knowledge, may not produce meaningful learning gains (Andrews et al. 2011).

There have also been a range of documented barriers to implementing active learning that span from individual instructor beliefs to course, departmental, and institutional level factors (Olmstead et al. 2026). For example, one of the most salient anecdotal and empirical barriers to active learning is student resistance, as faculty have noted challenges with student buy-in toward active learning activities despite the documented benefits (Felder 2011; Owens et al. 2020; Seidel and Tanner 2013; Shekhar et al. 2015). Structural constraints within large lecture courses include inadequate physical spaces to implement group activities (Olmstead et al. 2026), challenges with facilitating activities that are accessible to all students (Gin et al. 2020; Pfeifer et al. 2023; England et al. 2017; Cooper et al. 2018; Downing et al. 2020; Hood et al. 2021), and a lack of sufficient time to cover course content (e.g., Lund and Stains 2015; Shadle et al. 2017; Borda et al. 2020). Instructors have articulated their own anxieties with implementing new instructional approaches that they perceive may negatively impact their image and promotion prospects, which are further exacerbated with departments and institutions offering limited incentives to change, lack of supportive colleagues to enact this change, and insufficient professional development support (e.g., Andrews et al. 2015; Michael 2007; Walczyk et al. 2007; Ebert-May et al. 2011; Brownell and Tanner 2012; Lund and Stains 2015; Gehrke and Kezar 2017; Olmstead et al. 2026). These challenges may contribute to heterogeneous patterns of adopting active learning that have inhibited progression toward these practices becoming more normative in undergraduate biology education.

As active learning practices and challenges have been foregrounded in national conversations, discourse and research studies have largely centered around implementation of these practices in large, in-person courses. While the number of online courses in biology has been historically rarer (Varty 2016), the proportion of students taking at least one online course has steadily increased since 2019 across all disciplines, with a large increase during the COVID-19 pandemic (National Center for Education Statistics 2025). Although enrollment in online courses has since declined, the proportion of students taking these courses remains substantially higher than prepandemic levels.

Prior to the COVID-19 pandemic, the literature on online teaching largely focused on course design, faculty barriers, and student engagement (e.g., Lloyd et al. 2012; Zawacki-Richter and Naidu 2016; Wingo et al. 2017; Martin et al. 2020). The pandemic then required higher education instructors to rapidly pivot to online remote teaching with little preparation for adapting their in-person teaching activities to an online context. This period was rife with challenges for instructors, especially those who tried active learning strategies that were not easily adaptable, resulting in new approaches and challenges to sustaining constructivist learning experiences (e.g., Harris et al. 2020; Morrison et al. 2021; Rossi et al. 2021; Zuckerman et al. 2021; Durham et al. 2022). These challenges were compounded by barriers such as unequal access to technology, along with strains on work–life balance and the mental and emotional health of students (Durham et al. 2022). Beyond these immediate barriers, the pivot to remote instruction may have had lasting effects on students' orientation toward active learning. A longitudinal study on student perceptions of active learning found that student familiarity with active learning decreased during emergency remote instruction and remained lower than prepandemic levels, which may have resulted in exacerbated student resistance following emergency remote instruction (Craig and Hsu 2025).

Even though the number of published studies on remote learning increased during the COVID-19 pandemic, the literature on implementation of active learning strategies in online undergraduate biology courses remains nascent, and there is still a lack of standardized guidance for effectively integrating these practices into online biology courses for many instructors. Prior work exploring active learning in online courses identified the need of a robust set of design principles for implementing active learning and increasing student engagement in online courses (Khan et al. 2017; Chen et al. 2018) and the need for additional faculty professional development efforts to support online active learning comparably to in person instruction (Miner-Romanoff et al. 2019). There have been a few studies that directly compared student outcomes using active learning between in-person and online courses. For example, multiple studies have compared exam scores and course grades in online and in-person courses with active learning interventions, with several noting comparable performance across both modalities depending on course structure (e.g., Gavassa et al. 2019; Foster-Hartnett et al. 2022; Bonney 2024). Other studies have examined student perceptions of active learning across modalities (e.g., Beimel et al. 2024; Bonney 2024)—for example, one study found that in-person discussions were perceived as fostering stronger community while online courses were perceived as offering more opportunities for crafting stronger evidence-based arguments (Garces et al. 2024). However, there have been few studies that have directly compared instructor experiences with active learning practices across modalities.

Biology instructional reform is at a critical juncture in this period following the COVID-19 pandemic. As universities aim to reach larger populations of students, online and hybrid learning, where instructors deliver course material both online and in-person, will likely continue to be explored as an avenue for delivering biology content to more students (Cheslock and Jaquette 2022; National Center for Education Statistics 2025). Because the rapid shift to remote teaching has created a sizable group of instructors who have now taught biology courses online, there is a timely opportunity to learn from their firsthand experiences implementing active learning in this modality and compare them to their in-person course experiences. Examining instructors’ experiences using active learning is advantageous as online instruction becomes more widely institutionalized, especially as instructors who already use active learning in person offer a valuable comparative basis to understand what adjustments were needed to adapt these strategies online. It is important to use this time to document lessons learned from these experiences and consider scalable strategies for adapting active learning approaches to online biology instruction for instructors who may need to make a similar transition.

To address this need, we conducted a national exploratory study of undergraduate biology instructors who have implemented active learning in both in-person and online learning contexts. This study addresses the following research questions:

RQ1. What are the experiences of instructors who implement active learning teaching strategies in online vs. in-person college biology classrooms?
RQ2. What advantages and barriers do instructors report encountering when implementing active learning in online vs. in-person college biology learning environments?

We document these experiences to offer recommendations for instructors and institutions seeking to scale online biology instruction with student-centered active learning pedagogical approaches.

## Methods

### Study context

Approval for this study was obtained from the Institutional Review Board at Cornell University (IRB # 0149350). To recruit a sufficient number of participants who taught both in-person and online/hybrid courses, we invited instructors who had taught one or more biology courses in both of these modalities during the time frame of 2019–2025, which encompasses course instruction before, during, and following the COVID-19 pandemic. An invitation to participate in the survey was sent out using snowball sampling (Patton 1990) and targeted recruitment. Participants were identified in two stages. First, we curated a list of authors who had published one or more lesson plans for online biology teaching for initial recruitment, as it was assumed that these instructors had taught at least one online course. This list was curated from a scoping review conducted by Treibergs et al. (2025), which identified peer-reviewed lesson plans published between 2000 and 2022 across several journals [e.g., *CourseSource, American Biology Teacher, SERC, Teaching Issues and Experiments in Ecology (TIEE*)]. To capture more recent lesson plans, we hand-searched the journals included in the scoping review for online/hybrid course lesson plans published between 2022 and May 2025. We sent a direct email to the first and/or corresponding authors of these lesson plans to invite participation.

Second, following this initial recruitment, we disseminated the survey more broadly on several list servers reaching college biology educators (e.g., Ecolog-L, the Society for the Advancement of Biology Education Research). We also reached out directly to department contacts at different institutions, with the goal that institutions would disseminate the survey to potentially qualified participants within their departments. This recruitment strategy was aimed at recruiting instructors from a variety of institutions, including 2-year and 4-year colleges. The goal was to recruit between 50 and 100 instructors to obtain a sample size that was suitable for the exploratory nature of this study. We administered the survey via Qualtrics (Provo, UT) and included screening questions to identify respondents who met criteria for participation. To ensure that prospective participants had a consistent interpretation of active learning when evaluating the courses they taught, we used the definition provided from Dreissen et al. (2020). Survey respondents must have taught both the online and in-person courses using active learning strategies that aligned with this definition, and instructors were required to have taught at least 25% of the total course time (e.g., for cases where the course was co-instructed). Respondents who did not meet all criteria were prompted to exit the survey. All respondents who were eligible and completed the full survey received a $20 incentive. Here we analyze responses from 79 instructors.

Survey respondents were prompted to indicate whether the online course modality was asynchronous, synchronous, or hybrid (Fig. 1): the most common mode was synchronous. We included instructors of hybrid courses in this study because they involve substantial online instructional design, offer relevant insights for our comparison to fully in person courses, and can be an alternative to fully online or in-person courses (Allen et al. 2007; Vaughan 2007). Respondents who selected “other” for their online course modality primarily stated that the course was a mix of both asynchronous and synchronous online learning.

**Fig. 1:**
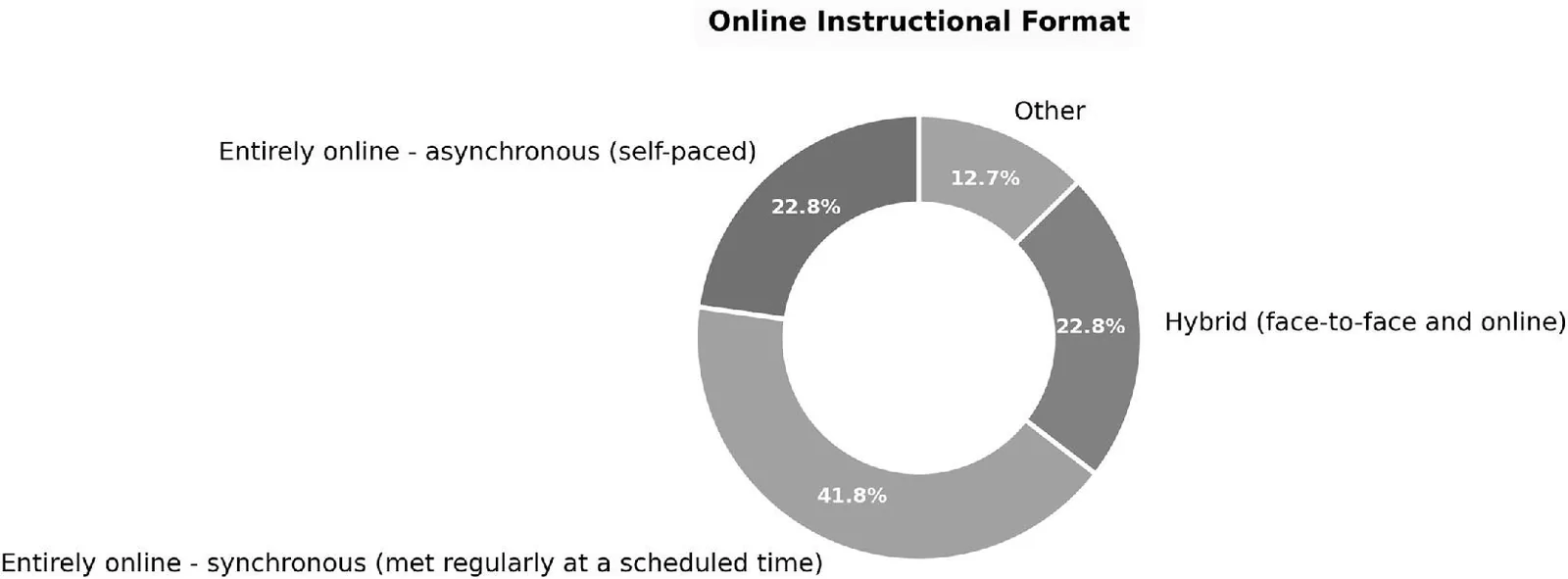
Disaggregation of instructional format of the online courses taught by instructors in the final survey sample (*n* = 79).

We classified institutional characteristics for respondents' online courses based on the Carnegie Classification of Institutes of Higher Education (Fig. S1) (McCormick and Zhao 2005). Based on our targeted recruitment efforts, we received responses from a variety of institutions. While the majority of instructors taught at doctoral-granting institutions, there was also representation of masters, baccalaureate, and associates granting institutions. A majority of instructors taught at institutions with high or very high research activity. Nearly half of the instructors taught at higher access institutions which serve larger proportions of students who receive Pell Grants and who identify with underrepresented racial and ethnic backgrounds based on geographical location. More than one-third of the instructors taught at minority serving institutions, classified using the Office of Postsecondary Education (ED) through the 2025 Eligibility Matrix (Rutgers Center for Minority Serving Institutions, 2025). We documented the teaching roles and experience of the respondents for both the in-person and online courses they had taught (Fig. S2). Most survey respondents are faculty members with several years of teaching experience.

### Instrument

The survey was designed to measure instructors’ attitudes toward implementing active learning practices for in-person and online/hybrid course modalities, as well as to explore the challenges and opportunities associated with active learning specifically in the online/hybrid modality. To the best of our knowledge, there are no standardized instruments that have been developed to systematically compare instructors experiences with implementing active learning in both in-person and online courses, although there have been some instruments developed to broadly measure instructors’ perceptions of barriers associated with active learning for in-person courses (e.g., Pundak et al. 2009; Carroll et al. 2023; Sturtevant and Wheeler 2019). Here, we developed a survey to address our research questions that are guided by the challenges and opportunities associated with implementing active learning in both in-person and online modalities previously identified in the literature (e.g., Olmstead et al. 2026). We view this survey as an exploratory tool to address our novel research questions rather than a reproducible, theoretically-aligned tool intended for broader use to measure active learning implementation in different contexts.

The survey separately measures attitudes toward learning in both online and in-person contexts. The items are paired to compare instructor experiences of implementing active learning within each modality. For example, survey respondents would answer: “Active learning was compatible with the way I liked to teach in my *in-person course*” and then “Active learning was compatible with the way I liked to teach in my *online course*.” For each item, respondents were prompted to reflect on their most recent course that they had taught in that modality when selecting their rating; the in-person and online courses they considered could have been either the same course or different courses. For each item, respondents were prompted to rate on a scale from 1 = Strongly Disagree to 7 = Strongly Agree. Initially the survey consisted of 25 Likert-scale items, but five negatively worded items were removed due to concerns that the shift in valence may have obscured participant response patterns. Each of the remaining 20 items was replicated to measure attitudes toward learning in both online and in-person contexts separately, resulting in a total of 40 items that were analyzed.

To provide additional insights that could potentially complement the trends observed in the quantitative items, we asked three open-ended questions. The questions prompted respondents to reflect on their online course and consider (1) barriers to implementation of active learning practices, (2) active learning practices that worked *better* in an online setting, and (3) advice that could guide instructors for integrating active learning strategies into online biology courses. These additional questions were included to leverage participant voice and reflections to offer actionable strategies for instructors who are considering active learning for online course instruction.

### Data analysis

#### Quantitative analysis

We conducted cognitive interviews to generate content validity evidence to ensure respondents were interpreting the survey items consistently. Four interviews were conducted with instructors and discipline-based education researchers familiar with both in-person and online teaching in higher education biology, and changes in item wording were made based on iterative feedback. Our analysis focuses on the item level to describe patterns rather than infer latent structure based on these constructs because these items were not derived from a single theoretical perspective and our sample size is limited. Collectively, measures of these items provide a descriptive exploration of variation in instructors’ experiences with active learning in online and in-person courses.

We conducted statistical analyses using the open-source programming environment Python. Claude.ai was used to assist with figure and code development, which was validated by the authors. We calculated descriptive and inferential statistics for each item measured in the survey. Because of assumed non-normality due to the ordinal nature of the items, we calculated a Wilcoxon signed-rank test for each item as a paired sample comparing participants’ relative perceptions of implementing active learning in in-person and online course modalities. A Holm-Bonferroni correction was then applied to control for the Type I error rate for multiple comparisons. Effect size was calculated for each item using Rosenthal’s R (Rosenthal and Rubin 2003), with the following cutoffs: small = 0.1–0.3; medium = 0.3–0.5; large > 0.5.

Because Likert-scaled items are ordinal and participant responses were skewed toward higher ratings for a majority of the items, we analyzed item medians and interquartile ranges to categorize commonalities and variations in instructor experiences with active learning across their online/hybrid and in-person courses. Thirteen of the twenty items aligned with three categories, and inferential statistics for all items are summarized in Table S1.

#### Qualitative analysis

The three open-ended questions were analyzed using inductive coding. Codes were developed through an iterative reading of the open-ended response questions (Saldaña 2021). A separate codebook was generated for each question, and the inductive codes were then applied to each participants’ response. Multiple codes could be applied to each response, as some respondents articulated multiple barriers or strategies that worked better in online active learning settings. We calculated the frequency of the inductive codes for the first two open-ended questions to indicate salience of barriers and relative advantages of implementing active learning strategies in online courses. We also disaggregated the frequency of codes by online course format (asynchronous, synchronous, and hybrid) and relative proportions were calculated within each format.

ALZ served as the primary analyst, generating the codebook for each open-ended question. Having a single researcher develop the codebook is common in qualitative content analysis to ensure a consistent frame of reference in which codes are defined and applied, given that there are post-hoc steps taken to ensure the codebook reliably represents the experiences of the participant sample (Schreier 2012; O’Connor and Joffe 2020). A subset of the responses (∼20%) were then coded by MKS for the first two questions, and the two researchers had met to resolve disagreements and refine code definitions until dialogic consensus was reached. MKS did not identify any emergent codes that were needed beyond the existing codebook, and the two coders achieved substantial interrater agreement of 95.3% and a Cohen’s kappa of 0.75 (Cohen 1960; Landis and Koch 1977). Given the high inter-rater agreement between the two coders, ALZ applied the codebooks to the entire data sample. For the third question that had more codes that were applied at a lower frequency relative to the other open-ended questions, ALZ and MKS engaged in peer debriefing by discussing examples of each code to reach consensus on code definitions and boundaries (Lincoln and Guba 1985). Finally, we shared the coding scheme and applications to the data in research team meetings and at a regional conference to researchers external to the project. We used iterative feedback obtained from those sessions to further validate that the claims being made were defensible and grounded in the data.

## Results

### Comparing experiences active learning in in-person and online course modalities

We compared instructors’ experiences implementing active learning in online and in-person courses using pairs of survey items designed to measure the same factors across both modalities (Table S1). Instructors largely rated items for their in-person courses higher than their online courses. Here, we summarize the items that fall into three categories.

#### Active learning is perceived as beneficial for student learning and engagement in both online and in-person courses

Instructors reported *active learning as beneficial* in both their in-person and online courses (median of 6 or 7 in both modalities) (Fig. 2). Instructors generally reported that active learning was compatible with their teaching preferences and intended student learning outcomes both in-person and online. Across both modalities, respondents perceived that active learning was more effective at promoting student learning *and* engagement, assisting instructors in identifying instances where a concept was not well understood, and making their job feel overall more enjoyable than lecturing.

**Fig. 2:**
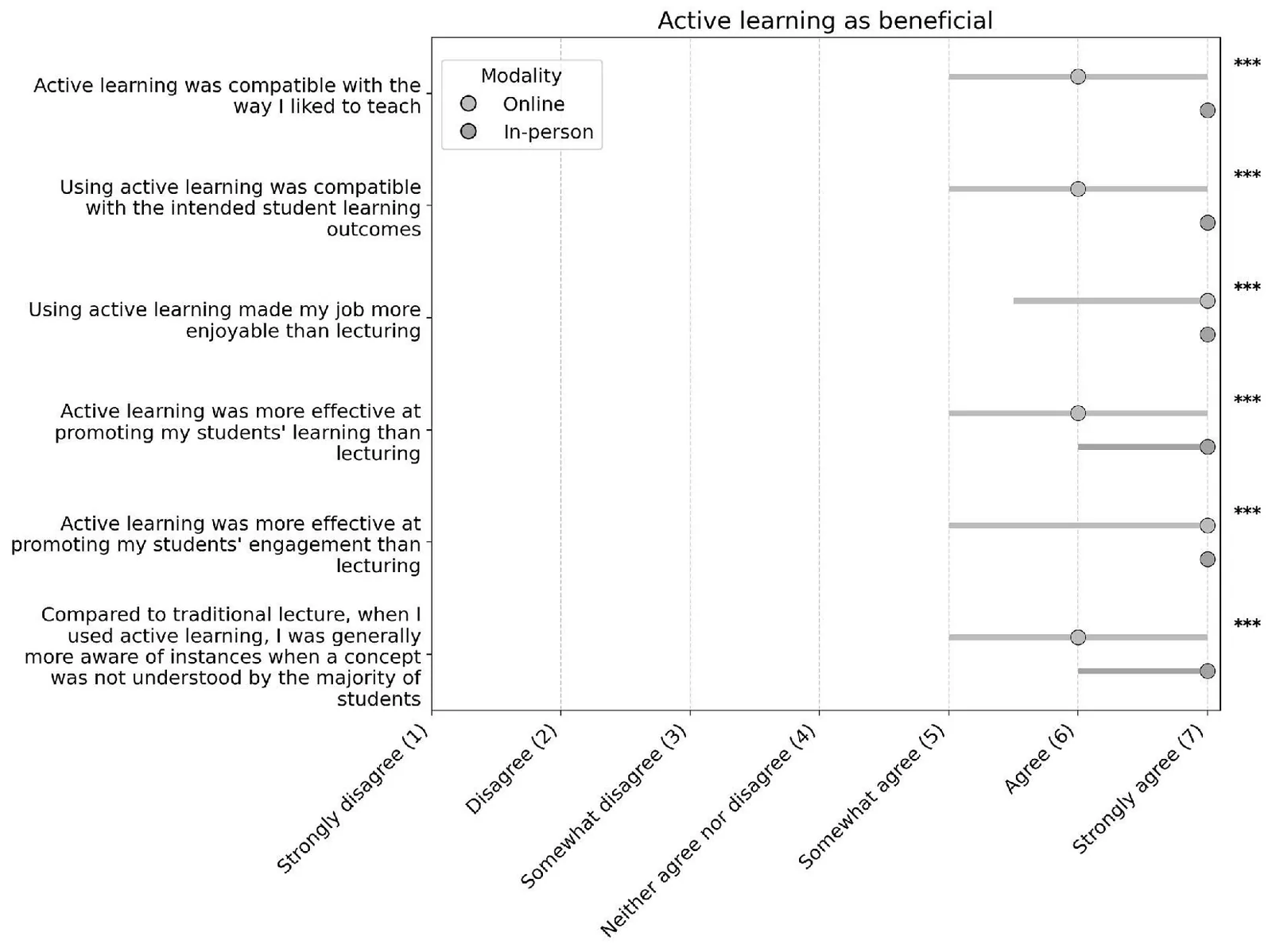
Items where active learning was perceived as beneficial in both in-person and online contexts. The median of these items was at least “Agree” (6), with a first quartile of at least “Somewhat agree” (5). Medians are represented by dots, and the third and first quartiles are represented by the upper and lower edges of the dot plots. *** indicates p < 0.001.

#### Less departmental and collegiate support of active learning in online courses

The largest differences in experiences of implementing active learning between online and in-person modalities (absolute median difference of 2 or higher) related to *departmental and peer support* (Fig. 3). Instructors perceived that their department head encouraged them to use active learning strategies and were recognized by others in their department for using active learning to a stronger extent in their in-person course compared to their online course. Similarly, instructors reported greater exposure to departmental support of active learning in their in-person courses, both in terms of observing colleagues implement active learning strategies before adopting and engaging in departmental discussions about implementation.

**Fig. 3:**
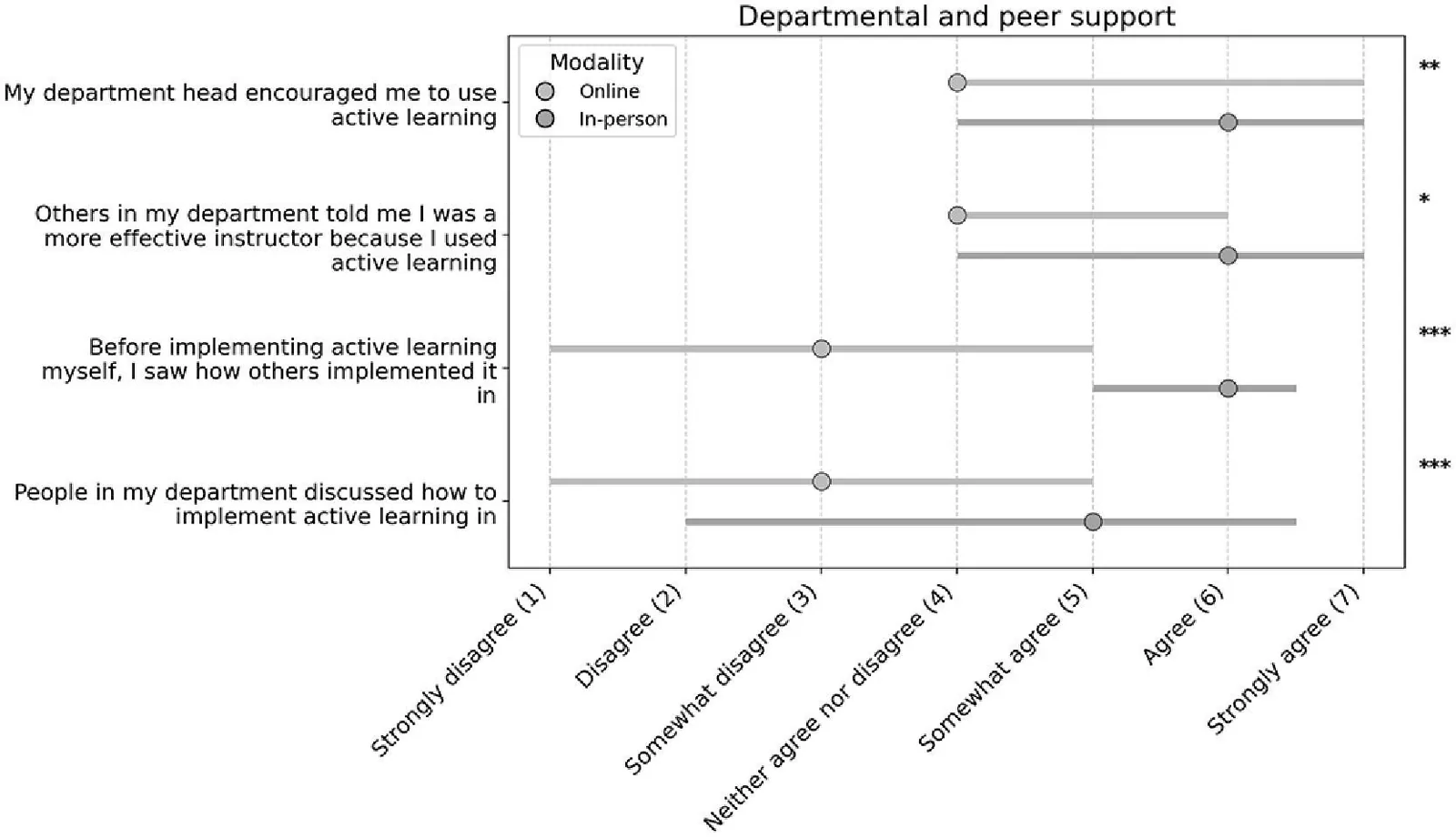
Items where active learning was perceived as different between in-person and online modalities related to departmental and peer support. The absolute median difference between these items is at least 2. Medians are represented by dots, and the third and first quartiles are represented by the upper and lower edges of the dot plots. * indicates p < 0.05, ** indicates p < 0.01, and *** indicates p < 0.001.

#### Large variations in resource availability within modalities

The responses to several items demonstrated that active learning implementation had a substantially larger interquartile range in either the in-person or online modalities (Fig. 4). These differences were in relation to the *availability of resources* for implementing active learning. While instructors generally perceived that they had access to appropriate resources for implementing active learning, there was substantially higher variation in these ratings for their online courses.

**Fig. 4:**
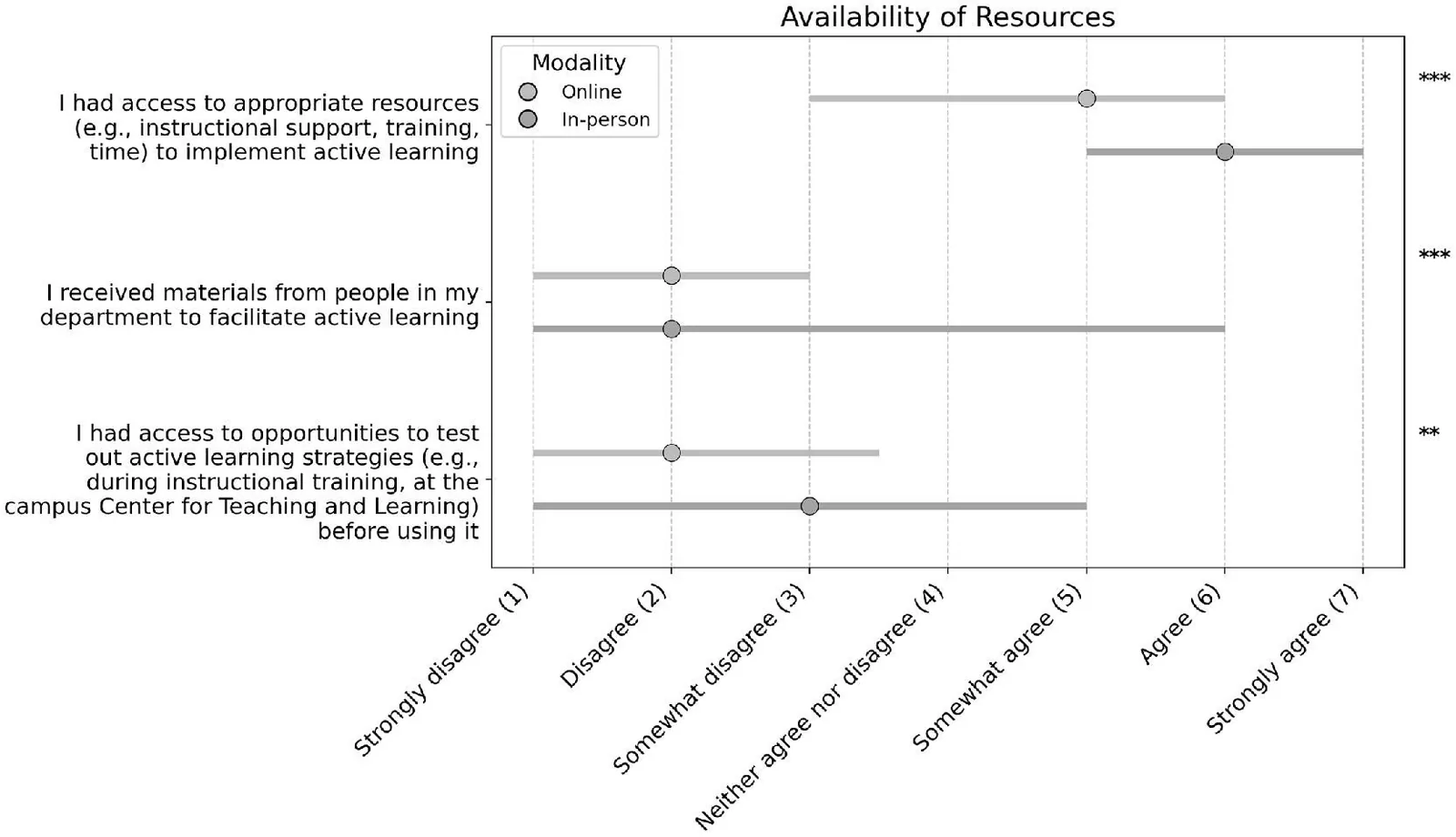
Items related to resource availability had higher interquartile ranges in one of the modalities. Medians are represented by dots, and the third and first quartiles are represented by the upper and lower edges of the dot plots. ** indicates p < 0.01 and *** indicates p < 0.001.

In both modalities, instructors generally disagreed that they had opportunities to receive materials from others in their department to facilitate active learning and were unable to test out active learning strategies through substantial training initiatives prior to implementation. However, the larger interquartile range in the in-person modality suggests that a meaningful subset of instructors did have access to these supports for their in-person courses.

### Barriers to implementing active learning in online courses

While active learning strategies were generally perceived as favorable for improving instructor’s teaching practices in both their in-person and online courses, the qualitative reflections on the barriers to implementing active learning add nuance to these emerging trends. While participating instructors largely perceived that implementation of active learning was more effective at supporting student engagement than lecturing in both in-person and online courses, nearly 40% (*n =* 30) of respondents identified *student engagement* as a salient barrier to implementing active learning in their online courses (Table 1). These barriers were cited at a higher relative proportion for synchronous online and hybrid courses, where students were participating in active learning activities in real time (Table S2). The respondents attributed challenges in student engagement to lack of student buy-in or unwillingness to participate in whole class discussions or peer to peer learning. Respondents reflected on observable behaviors that suggested lack of engagement, such as students not turning on their cameras or microphones, refusing to engage in breakout room discussions during synchronous sessions, or minimally engaging in asynchronous course activities.

**Table 1:** Code frequencies (codes with frequency >1) for barriers to implementing active learning in online courses that are not typically present in in-person courses.

Code	Definition	Number of responses	Sample response
Student engagement	Challenges with student buy-in or willingness to participate in active learning activities	30	“Student buy in!! I did not see a single camera on during class hours throughout the whole online semester. When polled, students reported that only 50% of breakout room time was actually productive (I.e. classmates actually unmuted and talked).”
Monitoring or receiving feedback on engagement	Receiving student feedback or assessing whether students are engaged in activities	19	“Although breakout rooms are great, it’s hard to hear if the whole class is “getting stuck” simultaneously. That is an advantage of the classroom is immediately collecting everyone to revise instructions, review a problem together.”
Inconsistent participation times	Students not participating in activities synchronously (e.g., in different timezones)	11	“Time zones and not being physically in the same space are the greatest barriers to implementing active learning in an online course. These limit the types of active learning strategies you can utilize and what you can expect from students regarding activities.”
Technological barriers	Challenges with students or faculty using or accessing technology	10	“In zoom classes, the increased bandwidth from breakout rooms meant that some students would get booted off the call. I work in a rural area and internet connectivity was a major barrier for students, which meant that participating in active learning online was complicated for some students.”
Facilitating group work	Challenge with implementing group work effectively	8	“Also in section, students were put in breakout rooms, but the time it takes to transition students in and out of breakout rooms is longer than in-person, and students are much less likely to participate in breakout rooms.”
None	No barriers identified	6	“Asynchronous online environments provide a barrier to active learning to the same level as in-person or synchronous online course.”
Lack of resources and training	Insufficient guiding resources and training opportunities for implementing online learning activities	6	“Far fewer people had taught online so there were fewer examples available online and no one I knew was able to offer advice. I definitely felt the lack of a support network to discuss ideas.”
Rushed transition	Limited time to pivot to online learning (particularly due to the COVID-19 pandemic	6	“Generally it was the speed with which we transitioned to all online teaching during the pandemic. It was just “all of a sudden” we are working in that environment. Time was the barrier!”
Adapting in-person resources	Challenges with adapting in person activities to online setting	4	“I taught an online section twice with synchronous sessions and a large class. It’s logistically difficult to conduct some of the active learning activities that I can do efficiently and effectively in-person. For example, I always used clicker questions coupled with think-pair-share, and it’s very difficult to do the “pair” piece in Zoom. Any breakout room activity would be time consuming and impossible to monitor.”
Time commitment	Amount of time required to invest in online course development was significant and limiting	2	“The biggest barrier was time—it takes a lot more time, effort, and creativity to develop meaningful activities than it does to make PowerPoints. Most of this extra time was not compensated.”

There were also many cases where instructors described challenges related to *monitoring or receiving feedback on engagement* (*n =* 19). For example, while instructors could monitor breakout sessions individually, the inability to monitor the entire classroom simultaneously made it difficult to assess student understanding and reconvene intermittently as an entire class. This barrier was particularly pronounced for larger class sizes. As one instructor stated, “*in an in-person class, you’d be able to at least get a vibe of how engaged all the groups are, even if you can’t stop by each group individually*” yet the siloing of student groups to online breakout rooms inhibits this structural advantage.

The next two most frequently described barriers were *inconsistent participation times* (*n =* 11) (Table 1), primarily in asynchronous course structures (Table S2), and *technological barriers (n =* 10) for both students and instructors. Several instructors stated that asynchronous activities were included in their course design to accommodate students participating in different timezones, yet such a structure limited the range of active learning strategies that could be implemented. For example, several respondents articulated that it was more challenging to facilitate meaningful discussion forums that encouraged peer interaction because timezones did not align or students would wait until the assignment deadline to contribute, which reduced opportunities for real-time discourse. Several respondents across synchronous, asynchronous, and hybrid course formats reported insufficient technical support and training for interactive tools and observed that students’ inconsistent access to reliable, high-bandwidth internet further constrained their ability to participate equitably in course activities.

The participant pool included instructors whose impetus for teaching an online course was the onset of the COVID-19 pandemic, where they had to rapidly pivot to remote teaching without any prior planning or preparation. Not surprisingly, several instructors described insufficient time (*rushed transition*) to adapt in-person active learning activities to the online modality as a challenge with that transition (Table 1). Several instructors additionally noted insufficient resources to support online instruction, such as a lack of formal professional development and training on best practices, as well as the relative lack of available educational materials. These comments parallel the weaker sense of departmental support and resource sharing trending from the quantitative survey items (Fig. 3).

Additional patterns of barriers related to the quality of the group interactions that could be facilitated during synchronous online sessions (Table 1). Some instructors noted challenges with facilitating group work; for example, the transition into forming groups took substantially longer during synchronous sessions and the accountability for students to interact in groups for asynchronous activities was not feasible. A smaller subset of respondents did not identify any unique barriers to using active learning strategies online compared to in-person courses. Some of these respondents directly described the barriers as analogous to barriers of implementing active learning strategies in large, in-person lecture courses (e.g., challenges of facilitating group work in-person is similar for whether in a physical classroom or online breakout rooms).

### Active learning strategies that worked *better* in online courses

While a majority of respondents identified one or more barriers to using active learning in their online courses, they were further prompted to identify specific active learning strategies that worked *better* in their online course compared to their in-person course. Aligning with the high frequency of barriers identified, a substantial number of respondents (*n =* 21) stated that there were *no active learning strategies* that worked better in their online courses compared to their in-person courses (Table 2), with a notably higher relative proportion in asynchronous courses (Table S3).

**Table 2:** Code frequencies (codes with frequency >1) for active learning strategies that worked better in online courses compared to in-person courses.

Code	Definition	Number of responses	Sample response
No relative benefits identified	No activities identified as more advantageous for online active learning	21	“Any active learning activities that can be done asynchronously, they can be as effective in online setting as in in-person setting. I cannot think of any active learning strategies that work better in online settings than in in-person settings, at least not in my large classes (hundreds of students).”
Group work via breakout rooms	Group work or small group activities, likely using breakout rooms	15	“Regrouping at key intervals for discussion during group work can work better in the online environment as reminders can be easily sent as announcements (for instance, “you have one more minute before we regroup”), and when the breakout rooms close, students have to come back in the main room and can’t continue talking together in the same manner they can in an auditorium.”
Polls	Zoom or other interactive polls	9	“I use a lot of polls as formative assessment in both online and face to face classes. I think polls actually work better in a synchronous online setting, rather than a face to face setting. I’m guessing nervousness is the reason why.”
Chat log/blasts	Using the chat feature to record responses or encourage mass participation	7	“The only engagement strategy that works better online than in-person is the zoom chat, which doesn’t exist in an in-person setting. But I loved being able to get real time feedback from students that was private, they only messaged directly to me. I can’t think of anything else that is better online than in-person.”
Collaborative documents	Activities where multiple people can collaborate on a single document	6	“Collaborative authoring of short answer questions (like in a google doc) is easier/better online, because the students are only focusing on one thing and can work independently but together. That works well if the questions only require text answers (not drawing or graphs).”
Discussion boards	Use of online discussion boards or forums	5	“Discussion boards seemed well-suited to my online, asynchronous course. Students could do the readings when convenient to them and write when they had time. They had to respond to at least one other student, which would be more chaotic in-person than online.”
Engagement of shy students	Activities that are easier for students who may be resistant to participating	3	“I do think that requiring discussions and requiring interaction among students does lead to more class-wide engagement/participation online than it does in-person because shy students that may be afraid to speak up in class have an opportunity to reflect and carefully craft their statements.”
Case studies/demos	Working on case study examples or demonstrations in more depth	3	“I’ve found that strategies that require some research and reading work better online, such as disease case studies. In class it’s more difficult for students to find reliable sources on their phones vs at home on a tablet/computer, and I do not require them to bring a device to class.”
Interactive modules	Computer-based activities or modules	3	“Paper discussions using Feedback Fruits often works better and has more student interaction in the LMS than in-person. I found this surprising and have moved a few discussions for the in-person class to the LMS.”
Activities requiring extended time	Activities that require a significant amount of time	2	“Exercises that took longer to complete could be accomplished in an online environment. My students make observations in nature and online students were able to explore different placed compared to in-person students that are limited to campus.”
Community based projects	Projects that allowed students to interact with their broader communities in some capacity	2	“A personal project that students can complete individually, such as conducting research in their own residential community, because it is both accessible and familiar to them.”

The *use of breakout rooms* was the most frequently described active learning strategy that worked better in the online course setting (*n =* 15), with higher relative proportions for synchronous and hybrid courses (Table 2 and S3). When functioning as intended, the smaller setting of the breakout room was conducive for productive discussion without distraction from high noise levels typically observed in a large in-person course. Respondents who reported this advantage found that organizing the groups and reconvening at key intervals as an entire class was generally easier given the built-in functionality of the platform to manage the creation and transition into breakout rooms.

Other respondents stated that a primary advantage of the online modality was that several active learning strategies are better suited for students who would otherwise be reluctant to participate in a large-group setting (Table 2). These included high participation strategies such as using the platform chat and polling systems, which were perceived as less susceptible to student resistance. Respondents praised the use of the chat feature for allowing more synchronized participation among all students (e.g., prompting students to wait to hit “enter” to submit their responses simultaneously) or to anonymously message the instructor to provide real-time feedback.

Another advantage to online active learning was the implementation of strategies that allowed for more in-depth exploration and discussion (Table 2). Several respondents described the increased feasibility of case studies that typically had a research or reading component that could be partitioned over multiple class periods or activities that allowed for more community-building and individual student creativity. Respondents frequently described asynchronous discussion forums as more successfully implemented in their online courses, given that this provided a rarer opportunity and motivation for students to interact with each other when there were limited face-to-face contact hours. Finally, synchronous collaborative activities using shared documents or virtual whiteboards were easier to implement online due to screen-sharing capabilities, which enabled students to contribute at a comfortable pace.

### Participant advice for implementing active learning in online courses

Participating instructors were prompted to reflect on the supports and resources that they perceive are most pivotal to implement active learning strategies more ubiquitously in the online context (Table S4). The coded responses had more variation in comparison to the first two open-ended questions, yet there were several salient patterns in advice, and the most common suggestions are presented in Fig. 5 and summarized here.

**Fig. 5:**
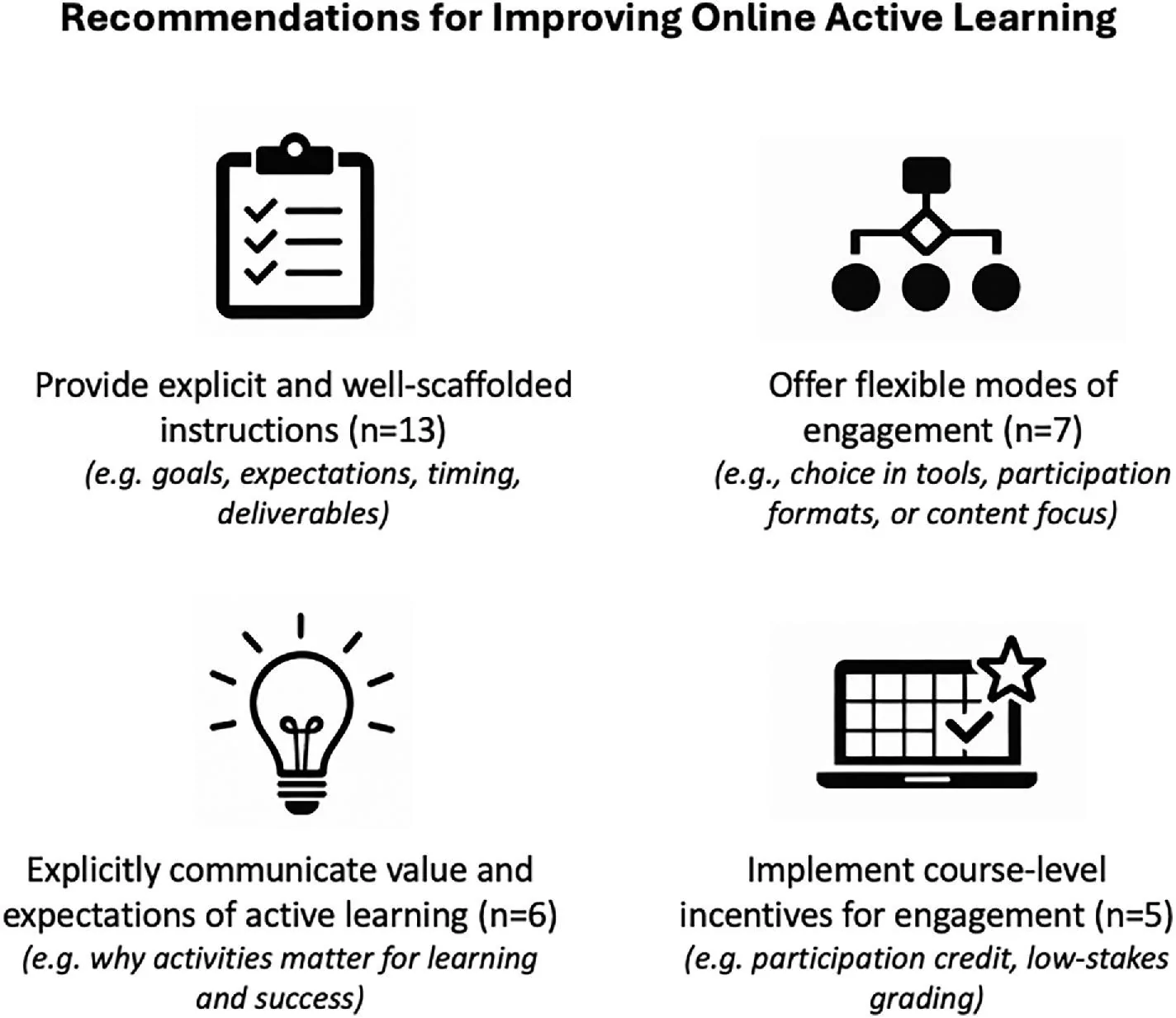
Most common recommendations for improving implementation of active learning in online biology courses. A full codebook is available in Table S4.

Several of the coded responses emphasized the need *for more explicit and clear instructions* to students when setting up an active learning activity, even more so than for an in-person course (*n* = 13). For example, one respondent stated that “*I think it is especially important with online students to be sure expectations are clear since you can’t course correct an activity in real time.*” Another respondent listed “*clear instructions, rubrics, guide videos, tutorial videos, introduction videos, examples of completed activities*” as needed to orient students to the types of activities they will encounter to help students prepare in advance so that class time can be used more effectively for active learning.

The second highest coded recommendation from participants was offering *flexible engagement options* in active learning activities to increase student motivation and make active learning more inclusive. These flexible options included allowing students to choose the tools to engage, “*like letting them use any software they wish to draw an image, or to use paper and pencil and take a picture to create a diagram*” or “*using a system (like Nearpod) that allows for the students to interact with the material in multiple ways (e.g., multiple choice, short answer, drawing).*”

Several instructors also shared the need for *emphasizing to students why active learning is especially important in the online context* and providing rationale for why certain active learning strategies are being used. They contended that establishing expectations early on includes informing students how they will be assessed for their engagement, as one instructor stated: “*I think setting expectations at the beginning of the semester is incredibly important. When I teach this course again, I think I will establish that I will be dropping into [Z]oom breakout rooms at random to answer questions and monitor progress. This may incentivize student engagement.*” Another instructor concisely noted, “*transparency is key to student buy-in.”*

To *incentivize engagement* at the course-level, several instructors noted the importance of integrating low-stakes, high-participation active learning strategies, such as polling, consistently during synchronous sessions. As one instructor stated*, “I might suggest using many low-effort, high participation strategies and planning these in advance. If these activities are well-placed, they can inject the class with more energy and serve as a valuable understanding check.”* They contended that *building credit for participation* in these activities into the course grading scheme should be considered to incentivize consistent and thoughtful effort in these activities.

Other advice that was coded at a lower frequency included encouraging students to turn their cameras on during synchronous class sessions, implementing consistent icebreaker activities, and developing more asynchronous community-building activities (Table S4). For example, a few instructors suggested the power of students creating short video explanations, peers then commenting on these videos, and then responding to feedback to bring the “human element” back into the asynchronous environment. A few instructors who used a “flipped classroom” approach (e.g., Bishop and Verleger 2013; Herreid and Schiller 2013; O’Flaherty and Phillips 2015; Jensen et al. 2015; Styers et al. 2018) described the implementation of both asynchronous and synchronous active learning techniques as synergistic and allowed for deeper student engagement and community. As one instructor shared: “*it allowed me to get to know the students but avoided the dynamic of me just talking into the void*.”

## Discussion

Situated in the existing literature, this study fills an important gap by exploring instructor experiences with active learning in undergraduate biology courses. Importantly, instructors participating in this study had experience implementing active learning in *both* online and in-person contexts, providing a comparative frame of reference for identifying the relative affordances and drawbacks of implementing active learning in their online courses compared to their in-person courses. Our findings reveal substantial agreement among instructors regarding the benefits of using active learning compared to lecture regardless of instructional context. However, the findings also highlight that implementation of active learning in the online context presents instructors with ongoing challenges related to aligning their pedagogical goals with the online format that differ in fundamental ways from in-person instruction.

### RQ1. What are the experiences of instructors who implement active learning teaching strategies in online vs. in- person college biology classrooms?

Our survey measured instructors’ perceptions of active learning in both in-person and online courses to understand their relative experiences and attitudes toward these instructional experiences. The relative advantage of active learning strategies compared to traditional lecture style practices was perceived strongly in both in-person and online courses (Fig. 2), so there continues to be little qualm regarding the efficacy of these evidence-based practices when implemented across instructional modalities, consistent with other studies (e.g., Auerbach and Schussler 2017; Auerbach and Andrews 2018; Archie et al. 2024). Instructors also perceived that active learning practices were compatible with their intended pedagogy in both modalities (Figs 2), yet they received weaker departmental support and resource sharing in their online courses (Figs. 3, 4). The lower ratings on these items for the online contexts suggest that many instructors had minimal exposure to concrete examples or conversations with colleagues about active learning practices prior to and during implementation.

Instructors in our study voiced that there could be additional opportunities to offer support for active learning in online instruction. An empirical study of online teaching in engineering education previously argued that the social recognition of active learning is needed to improve the adoption rate among engineering faculty (Bordel et al. 2024). The weaker departmental support in online courses could be due to the rapid transition with emergency remote instruction or because the relative number of instructors who teach online courses using active learning are less represented within a department. Evidence-based practices often spread when potential adopters can see them in action (Rogers 2003), and it has been previously shown that preparing faculty members to implement active learning in in-person courses can also be used to prepare instructors teaching in the online context (Basdogan and Birdwell 2023). Departments could consider developing faculty learning communities where active learning resources and strategies can be discussed for instructors of these courses while also making these practices visible to the broader department (Addis et al. 2013; Elliott et al. 2016).

### RQ2: What advantages and barriers do instructors report encountering when implementing active learning in online vs. in-person college biology learning environments?

The participant sample in this study was uniquely positioned, as participating instructors had firsthand experience implementing active learning in both in-person and online biology courses, including fully asynchronous, fully synchronous, and hybrid online modalities. Each of these pedagogical experiences offer unique sets of advantages and challenges; our survey strategy provided a comparative frame of reference that allowed them to evaluate the relative affordances and limitations of active learning online versus traditional large, in-person lecture contexts. We focus here on larger differences between in-person and online courses while acknowledging that there are potential differences with synchronous, asynchronous, and hybrid courses (Tables S2 and S3).

Instructors in our sample were prompted to reflect on the barriers and relative affordances of implementing active learning in their online courses compared to their in-person courses. Active learning strategies that were described as more effective online than in-person were generally a product of structural affordances to implementing the activity rather than a pedagogical innovation that was unique to the online context (Table 2). Activities like discussion boards, chat blasts, and collaborative group artifacts can also be implemented in-person, yet the online platform features often made these activities easier to manage and more scalable. As summarized in the collated advice provided by the instructors (Fig. 5, Table S3), the challenge is not as much as the strategies themselves but *how* they are implemented. Instructors perceived that active learning techniques require more attention to ensuring a seamless transition into the activity–especially as direct verbal communication with the class will be limited in breakout rooms–the instructions prefacing the activity likely need more intentional scaffolding than what is often necessary within an in-person course context. Instructors also contended that increasing student buy-in was not just about structuring the active learning activities but also giving students shared control over how they engage in the activities.

There were some notable contradictions that emerged in instructor experiences. For example, some identified breakout rooms as the most significant barrier to engagement while others praised them as the most effective active learning strategy. However, there also may exist unmeasured moderating variables such as class size and technical support provided by institutions that influence the success of implementation. Nonetheless, these disparities suggest that the effectiveness of online active learning may hinge less on the choice of tool or strategy itself and more on the surrounding instructional design.

The most salient barrier documented for instructors’ online courses was the lack of student engagement, especially for synchronous course sessions, which raises the question as to whether online barriers are fundamentally different or represent amplifications of existing tensions. The structural features of online environments such as limited nonverbal communication and increased student anonymity may intensify pre-existing resistance rather than create entirely new forms of pushback. A known challenge in online courses is student resistance to turn on their cameras, which can be attributed to factors ranging from poor internet connection to students’ concerns about their personal appearance (Castelli and Sarvary 2021). However, previous work also documented that students perceive that there are fewer opportunities to collaborate and more difficulties in sustaining motivation in online courses (Hollister et al. 2022).

Student engagement typically involves three dimensions: (1) effort willing to engage in an activity (behavioral), (2) interest and affective dispositions (emotional), and (3) investment in learning goals (cognitive) (Fredricks et al. 2004). Survey respondents generally discussed challenges with students’ observable behaviors, including not participating during group work (Table 1). It has been previously documented that both instructors and students perceive that it is important for participation in active learning activities to be a required rather than optional part of online courses (Van Wart et al. 2025), and several respondents in our study advised on incentive structures to improve students’ cognitive investment. Alignment across all three of these dimensions is essential for improving student engagement in online learning, and efforts focused only on observable participation or grading incentives may be insufficient without parallel attention to students’ emotional interest and motivation, such as community building and offering students choice in how they engage with course content. Many instructors noted that flexible engagement could give students autonomy to make choices about the content in alignment with the course learning objectives, such as selecting specific topics or case studies. For example, instructors may consider providing online versions of activities or case studies, organized by learning objective and topic, and allow students to pick one to earn credit.

## Implications

Grounded in the triangulated findings and collated advice from the participants, there are several actionable implications for instructors and institutions to disseminate active learning more ubiquitously in their online biology courses. Instructors in our study recognized that explicit communication of pedagogical rationale and evidence of the benefits of active learning become even more important in their online courses. For example, the use of the Transparency in Learning and Teaching in Higher Education project (https://www.tilthighered.com/) provides a framework for guiding students to a metacognitive understanding of how they learn, as well as resources and coordinated data for instructors and institutional leaders on how to improve assignments, curricula, and assessments in alignment with this framework. Shared evidence can include showing historical course data that document improvements in exam performance or reduced failure rates following the implementation of active learning, as well as findings from the broader literature (e.g., Freeman et al. 2014; Theobald et al. 2020). Instructors could also regularly articulate how students will be evaluated for their participation in active learning (e.g., contributing to breakout room discussions, completing shared documents or whiteboard activities).

Resource sharing and departmental role modeling was perceived to be less available for online courses, making it more challenging for instructors to have observable examples of implementing active learning strategies. Departments and institutions can consider connecting instructors to repositories where they can upload sample activities, lesson plans, rubrics, and tutorial videos to encourage cross-sharing of resources. Departmental workshops and peer mentoring programs that facilitate instructors exchanging strategies and collaboratively building shared resources may further reduce the individual burden of course redesign.

In addition, online instructors can use published lesson plans to see example lesson materials (Treibergs et al. 2025). Published lesson plans shared as Open Educational Resources (OERs) consist of materials such as lecture slides, assessments, and classroom activities to provide instructors with increased course development support. These resources are already growing for the biology education research community through platforms such as the J*ournal of Microbiology and Biology Education* and *CourseSource* (Ahmed et al. 2024). Instructors can engage with these resources so they do not feel as if they have to “reinvent the wheel” and invest a significant cognitive and emotional load into building new activity infrastructure from scratch. Longer term professional development opportunities and peer review and feedback on materials are supports that have been identified to be needed for broader sharing of OER resources (Flowers et al. 2024). Biology instructors could seek out OERs for observable examples of how active learning can be implemented in online and hybrid courses, which addresses a challenge reported by several instructors who struggled to adapt in-person activities to their online courses.

## Limitations and directions for future research

There are several limitations to this study that have inspired directions for additional research. First, our analyzed sample is limited to 79 higher education biology instructors who implemented active learning in both in-person and online course settings (Fig. 1). While we recruited instructors from a national sample, we acknowledge the potential of self-selection bias that may not universally represent the experiences of all instructors.

Second, because of the small sample size, we were unable to further disaggregate our findings and make comparisons in experiences based on instructor and institutional characteristics. Modeling factors such as course size, course level, and institutional type could be used to identify how attitudes and perceived advantages and barriers underlie attitudes toward using active learning across different instructional modalities. Given that our sample largely included instructors who published educational materials or were contacted through list servers that reached biology researchers and educators, many of these instructors likely had training or research experience in evidence-based active learning practices. Professional development experience in active learning pedagogical practices may influence instructors’ perceptions of both the relative advantages and potential barriers to implementation, so intentional sampling of instructors that have varying levels of training with evidence-based practices may uncover additional factors that influence the sustained use of these strategies. Longitudinal studies could further examine how instructors’ perceptions and implementation of active learning change as online teaching continues to develop and new technological tools are introduced.

Third, as this study explores the understudied nature of instructors’ attitudes toward active learning practices, there remains a gap in triangulation of both the instructor and student perceptions of active learning use in online contexts. For example, instructors consistently identified student engagement and buy-in as the most salient barriers to implementing active learning (Table 1), which reflects a well-documented tension between the positive learning outcomes associated with active learning and students’ resistance to these approaches (Owens et al. 2020). Future research that simultaneously examines instructor and student perspectives could help clarify the sources of this misalignment and identify instructional practices that effectively support student buy-in.

Fourth, this study focuses on implementation of active learning exclusively in biology online courses. Future work could investigate how attitudes toward these practices may differ in other STEM disciplines. Such studies are particularly important given that biology students typically take gateway courses in mathematics, chemistry, and physics, which may also increasingly explore online courses as a way to deliver course content to more students. While different disciplinary courses may face unique challenges in implementing online learning based on the contextual nature of the discipline, there is often overlap in instructional goals when implementing active learning practices that align with the epistemological underpinnings of constructivist teaching. Identifying these commonalities and variations while also considering interdisciplinary forms of active learning could contribute to an integrated model of effective online learning in STEM education. Cross disciplinary research could also elucidate whether challenges with student engagement of active learning in online environments reflect broader pedagogical concerns that transcend content areas.

Fifth, although the number of online courses in biology is growing across institutions, the impetus for many of the participating instructors in this study to teach their class online was the COVID-19 pandemic. Several instructors stated that the rapid transition to remote learning afforded them little preparation time, which created a significant barrier to the effective implementation of active learning in their online courses. Their experiences may reflect adaptations in response to an emergency situation rather than deliberate instructional design that are aligned with goals of constructivist teaching. This sample likely included faculty who were resistant to online teaching and had to involuntarily transition to emergency remote instruction, which may have introduced instructors with negative response bias toward online instruction into the sample. Future research could purposefully target instructors whose experiences extend beyond the legacy effects of pandemic-era remote teaching. For example, a recent study found that students’ cognitive and emotional engagement had rebounded in online courses due to intentional design (Wester et al. 2025). Nevertheless, including instructors who navigated this rapid transition in the current study remains valuable for considering the challenges and strategies involved in using active learning under constrained conditions that limit instructor agency.

Finally, the instrument used in this study does not have structural validity and reliability evidence for constructs aligned with one or more theoretical perspectives, which will be necessary for robust measurement that leads to more generalizable inferences. Future instruments could directly explore how communication channels (e.g., faculty learning communities) and social systems within departments influence implementation decisions, as well as examine the temporal stages of adoption to identify transition points where support or barriers most strongly influence decisions to sustain or abandon active learning in online courses. Perspectives such as transformative learning theory could be applied to better understand how the evolution of individual beliefs about evidence-based methods can influence changes to their practice (Siddiqui and Adams 2013).

## Conclusion

The findings from this study demonstrate that while active learning is a favored evidence-based practice for both in-person and online undergraduate biology courses, there is work to be done to optimize active learning implementation in online courses. The reflections and advice provided by our participant sample represent actionable strategies for instructors and institutions who are seeking to scale online biology courses. More robust infrastructure for resource- and strategy-sharing at the departmental and national level will be an important step forward in disseminating tried and tested active learning strategies that can be implemented in a variety of online undergraduate biology courses. These ongoing efforts will be imperative to reduce the burden on individual instructors and ensure that active learning in online biology courses is sustainable and confers the similar documented learning and equity outcomes that have been consistently observed for in-person courses.

## Supplementary Material

obag053_Supplemental_File

## Data Availability

The data underlying this article will be shared on reasonable request to the corresponding author.
