# Supplementary material for: “Am I Teaching into the Void?” Instructor Perceptions of Active Learning in Online Biology Courses
Source: Integr Org Biol. 2026 Sep 18;8(1):obag053. doi: 10.1093/iob/obag053 (PMC13631680; doi:10.1093/iob/obag053)
Supplement: Supplementary file 1 [file obag053_supplemental_file.docx]

**Figure S-1.** Institutional characteristics of online courses taught by instructors (*n*=79) in the sample. Institutional classifications were determined by using the Carnegie Classification of Institutions of Higher Education based on the respondents’ self-reported institutional affiliations. Under ‘research activity’ characterization, RCU= Research College and University; R1= Doctoral Universities – Very high research; R2= Doctoral University – High research activity. Institutions classified as higher access serve larger proportions of students who receive Pell Grants and who identify with underrepresented racial and ethnic backgrounds based on geographical location.

**Figure S-2**. Self-reported characteristics of instructor respondents (*n*=79) in final data sample, disaggregated by course modality.

**Table S-1**. Descriptive and inferential statistics for all items in the survey. Responses are 1=*Strongly disagree* to 7=*Strongly agree.*

| **Item*** | **Online Mean ± SD (Median [IQR])** | **In-Person Mean ± SD (Median [IQR])** | **p (Holm)** | **Effect size \|r\|**** |
| --- | --- | --- | --- | --- |
| Before implementing active learning myself, I saw how others implemented it in ___ | 3.34 ± 2.04  (3.0 [1.0-5.0]) | 5.43 ± 1.61  (6.0 [5.0-6.5]) | <0.001 | large (0.68) |
| Active learning was compatible with the way I liked to teach in my ___ | 5.76 ± 1.21  (6.0 [5.0-7.0]) | 6.77 ± 0.53  (7.0 [7.0-7.0]) | <0.001 | large (0.66) |
| I had access to appropriate resources (e.g., instructional support, training, time) to implement active learning in my ___ | 4.72 ± 1.73  5.0 [3.0-6.0]) | 5.87 ± 1.51  (6.0 [5.0-7.0]) | <0.001 | large (0.61) |
| Using active learning was compatible with the intended student learning outcomes in my ___ | 6.03 ± 1.19  (6.0 [5.0-7.0]) | 6.80 ± 0.49  (7.0 [7.0-7.0]) | <0.001 | large (0.58) |
| I was able to assess (formally or informally) that active learning supported student learning in my ___ | 5.22 ± 1.54  (6.0 [4.0-6.0]) | 6.01 ± 1.08  (6.0 [5.0-7.0]) | <0.001 | large (0.53) |
| I was able to assess (formally or informally) that active learning supported student engagement in my ___ | 5.32 ± 1.39  (5.0 [5.0-6.0]) | 6.13 ± 0.97  (6.0 [6.0-7.0]) | <0.001 | large (0.53) |
| People in my department discussed how to implement active learning in ___ | 3.38 ± 2.16  (3.0 [1.0-5.0]) | 4.47 ± 2.37  (5.0 [2.0-6.5]) | <0.001 | large (0.53) |
| With my colleagues, I communicated the benefits of active learning in my ___ | 5.10 ± 1.51  (5.0 [4.0-6.0]) | 5.86 ± 1.37  (6.0 [5.0-7.0]) | <0.001 | large (0.51) |
| Active learning was more effective at promoting my students' engagement than lecturing in my ___ | 6.05 ± 1.22  (7.0 [5.0-7.0]) | 6.61 ± 0.82  (7.0 [7.0-7.0]) | <0.001 | medium (0.49) |
| I received materials from people in my department to facilitate active learning in my ___ | 2.48 ± 2.02  (2.0 [1.0-3.0]) | 3.46 ± 2.36  (2.0 [1.0-6.0]) | <0.001 | medium (0.48) |
| Compared to traditional lecture, when I used active learning, I was generally more aware of instances when a concept was not understood by the majority of students in my ___ | 5.61 ± 1.51  (6.0 [5.0-7.0]) | 6.38 ± 1.00  (7.0 [7.0-7.0]) | <0.001 | medium (0.47) |
| Active learning was more effective at promoting my students' learning than lecturing in my ___ | 6.04 ± 1.16  (6.0 [5.0-7.0]) | 6.51 ± 0.77  (7.0 [6.0-7.0]) | <0.001 | medium (0.46) |
| Using active learning made my job more enjoyable than lecturing in my ___ | 6.09 ± 1.29  7.0 [5.5-7.0]) | 6.68 ± 0.79  (7.0 [7.0-7.0]) | <0.001 | medium (0.46) |
| My colleagues expected me to use active learning in my ___ | 4.76 ± 1.76  (5.0 [4.0-6.0]) | 5.37 ± 1.72  (6.0 [4.0-7.0]) | <0.001 | medium (0.46) |
| I was able to try implementing active learning at my own pace in my ___ | 5.13 ± 1.97  (6.0 [4.0-7.0]) | 5.90 ± 1.30  (6.0 [5.5-7.0]) | 0.002 | medium (0.42) |
| My department head encouraged me to use active learning in my ___ | 4.87 ± 1.73  (4.0 [4.0-7.0]) | 5.33 ± 1.84  (6.0 [4.0-7.0]) | 0.009 | medium (0.36) |
| I had access to opportunities to test out active learning strategies (e.g., during instructional training, at the campus Center for Teaching and Learning) before using it in my ___ | 2.51 ± 1.85  (2.0 [1.0-3.5]) | 3.25 ± 2.21  (3.0 [1.0-5.0]) | 0.015 | medium (0.34) |
| Others in my department told me I was a more effective instructor because I used active learning in my ___ | 4.92 ± 1.57  (4.0 [4.0-6.0]) | 5.23 ± 1.54  (6.0 [4.0-7.0]) | 0.024 | medium (0.31) |
| My students saw me as a more effective instructor when I used active learning in my ___ | 5.03 ± 1.42  (5.0 [4.0-6.0]) | 5.27 ± 1.58  (6.0 [4.0-7.0]) | 0.13 | small (0.20) |
| Using active learning was my choice (i.e., not something required by my department head or job description) in my ___ | 6.54 ± 0.97  (7.0 [6.0-7.0]) | 6.52 ± 1.00  (7.0 [6.0-7.0]) | 0.41 | small (0.09) |

** Each item stem was contextualized to both "online course" and "in-person course."*

*** Effect size interpretation, based on Rosenthal's R (|r|); small = 0.1–0.3; medium = 0.3–0.5; large > 0.5.*

**Table S-2.** Relative proportions of codes summarizing barriers to active learning in online courses within each online course modality (asynchronous, synchronous, hybrid) for codes with a frequency of at least 5 across the sample.

| Code | Asynchronous  (n=18) | Synchronous  (n=33) | Hybrid  (n=18) |
| --- | --- | --- | --- |
| Student engagement | 15.4% | 32.6% | 38.1% |
| Monitoring or receiving feedback on engagement | 15.3% | 25.6% | 14.3% |
| Inconsistent participation times | 19.2% | 7.0% | 4.8% |
| Technological barriers | 7.7% | 6.9% | 9.5% |
| Facilitating group work | 3.8% | 4.7% | 9.5% |
| None | 3.8% | 2.3% | 9.5% |
| Lack of resources and training | 11.5% | 4.7% | 0% |
| Rushed transition | 3.9% | 9.3% | 4.8% |

**Table S-3.** Relative proportions of codes summarizing active learning strategies that worked better in online courses within each online course modality (asynchronous, synchronous, hybrid) for codes with a frequency of at least 5 across the sample.

| Code | Asynchronous  (n=18) | Synchronous  (n=33) | Hybrid  (n=18) |
| --- | --- | --- | --- |
| No relative benefits identified | 38.1% | 18.9% | 26.3% |
| Group work via breakout rooms | 9.5% | 21.6% | 15.8% |
| Polls | 0% | 13.5% | 5.3% |
| Chat log/blasts | 0% | 13.5% | 0% |
| Collaborative documents | 0% | 8.2% | 0% |
| Discussion boards | 9.5% | 2.7% | 10.5% |

**Table S-4.** Code frequencies (codes with frequency >1) for advice offered to improve active learning in online courses.

| **Code** | **Definition** | **Sample Excerpt** | **Number of Responses** |
| --- | --- | --- | --- |
| More explicit and clear instructions | Providing more scaffolded instructions prior to the start of the activity | “I think that providing clear instructions and articulating why you are encouraging students to use active learning, and how you will assess their active engagement is even more critical in an online environment than it is in an in-person course.” | 13 |
| Flexible engagement options | Offering students multiple avenues to participate in activity or engage with course material | “Also, giving students lots of options to complete tasks (like letting them use any software they wish to draw an image, or to use paper and pencil and take a picture to create a diagram) can lead to better results than trying to impose the same tools on everyone.” | 7 |
| Emphasize importance of active learning | Establishing expectations and rationale for active learning to increase buy-in | “For active learning online, start early and set expectations for students on what active learning is. Also, use the TILT framework to help students know why they should utilize active learning online.” | 6 |
| Incentives for engagement | Offering incentives for meaningfully participating in active learning activities, such as course credit | “I found that some students will just log into the session and then walk away. I suggest offering incentives to motivate the students to stay focused throughout the class session, for example by giving them credit for participating in polls. In smaller class settings, it is also a good idea to call on each student to contribute something to the class.” | 5 |
| Adapt in person activities | Using existing active learning activities for in person courses and adapting for the online context | “First, try it - it works better than you might think.  Second, activities often need to be modified to work in the online setting compared to the in-person setting, especially to involve and engage asynchronous students.” | 4 |
| Not worth it | General sentiment that trying to implement active learning in online courses is not worth the time and effort. | “I only used total online learning during Covid and was very happy to get back into the classroom a year later. So were the students. Face-to-face learning is just so much better than online learning (I've heard this from many colleagues as well).” | 4 |
| Variety of activities | Implementing a variety of active learning activities and resources to maximize engagement | “Utilize as many resources and activities as possible. Try to have group interactions through discussion boards.” | 4 |
| Consistent groups | Assigning students to consistent groups throughout the term | “For group exercises during the lecture, I think it would be helpful to keep the same breakout groups for the whole semester and ideally assign them to include a mix of undergraduates and graduate students. It would also be a good idea to inform the students that they will have a certain number of minutes to discuss before they will be brought back to the lecture.” | 3 |
| Alternate assessment | Providing students with different forms of assessment to measure learning and feedback | “While the nods and confused looks may not be visible in online environments, online learning offers some benefits, including the use of educational technologies and connecting students across countries and time zones. One suggestion is not to measure "success" in the typical ways we measure in-class participation.” | 3 |
| Cameras on | Requiring students to turn their cameras on during synchronous course sessions | “My greatest suggestion is to somehow get students to turn their cameras on for breakout sessions. We had classes of several 100 and students would not talk to each other in breakout rooms which essentially prevented good active learning. :((( I think it might be easier if there are smaller courses and students cannot "hide" as well even online.” | 3 |
| Effort and reflection | General advice for trialing activities and iteratively reflecting on implementation | “Try it and do your best. Don't spend too much time worrying about how successful it was, but do reflect on ways to improve in the future.” | 3 |
| Polls | Implementing a poll system for mass student participation | “Use a poll response system like iClicker or poll everywhere to check in on the students & their learning.” | 3 |
| Online/company resources | Use of existing resources and technologies that are available for online courses | “Take advantage of online tools. Simulations, games, activities, work boards, collaborative documents, etc. These are easier to use in online courses.” | 3 |
| Keep activities relevant | Being intentional about what activities are included to align with course goals and learning objectives | “Similar to in-person, be intentional. Think about the purpose/goal/learning objective and if your activity is an effective way for students to learn that particular LO.” | 3 |
| Consistent icebreakers | Using icebreaker activities to build community in the online classroom. | “I would encourage community building at the beginning of class by asking some sort of ice breaker question.” | 2 |
| Expect hiccups | Adoption of a mindset that expects iteration and productive failure as part of the process | “Try things out and don’t be afraid if they don’t work well the first time.” | 2 |
| Flipped classroom | Strategic use of the flipped classroom model | “Get support from your local teaching center to iterat[iv]ely (not all at the same time!) switch to a flipped model. Work on 2-3 lessons at a time and after a few years, you flipped it!” | 2 |
| Increase offline opportunities | Use engaging asynchronous activities to engage students with their peers and communities | “Active learning activities that can be done asynchronously seem work better for me, such as structure authentic inquiry projects, creative projects (e.g., we had a "Picture an Ecologist" video assignment), peer reviews, structured online discussions, engagement of interactive learning materials with both formative and summative assessment built in (e.g., SimUText Ecology), and reflections.” | 2 |
| Students make video explanations | Students create videos of them explaining concepts and then interacting with other students’ videos | “I think students making videos and commenting on them could be helpful.” | 2 |
